# Bionomics and Spatial Distribution of Triatomine Vectors of *Trypanosoma cruzi* in Texas and Other Southern States, USA

**DOI:** 10.4269/ajtmh.17-0526

**Published:** 2017-11-06

**Authors:** Rachel Curtis-Robles, Sarah A. Hamer, Sage Lane, Michael Z. Levy, Gabriel L. Hamer

**Affiliations:** 1 Department of Veterinary Integrative Biosciences, Texas A&M University, College Station, Texas;; 2 Department of Epidemiology and Biostatistics, Texas A&M Health Science Center, College Station, Texas;; 3 Department of Biostatistics and Epidemiology, University of Pennsylvania School of Medicine, Philadelphia, Pennsylvania;; 4 Department of Entomology, Texas A&M University, College Station, Texas

## Abstract

Defining spatial and temporal occurrences of triatomine vectors of *Trypanosoma cruzi*, the agent of Chagas disease, in the US is critical for public health protection. Through a citizen science program and field collections from 2012 to 2016, we collected 3,215 triatomines, mainly from Texas. Using morphological and molecular approaches, we identified seven *Triatoma* species and report sex, length, and blood engorgement status. Many citizen-collected triatomines (92.9%) were encountered indoors, in peridomestic settings, or in dog kennels and represent spillover transmission risk of *T. cruzi* to humans and domestic animals. The most commonly collected species were *Triatoma gerstaeckeri* and *Triatoma sanguisuga*. Adult *T. gerstaeckeri* were collected from May to September, peaking from June to July, whereas adult *T. sanguisuga* were active later, from June to October, peaking from July to September. Based on cross correlation analyses, peaks of captures varied by species and across years. Point pattern analyses revealed unique occurrences of *T. sanguisuga* in north and east Texas, *T. gerstaeckeri* in south and west Texas, *Triatoma indictiva* and *Triatoma lecticularia* in central Texas, and *Triatoma rubida* in west Texas. These relatively unique spatial occurrences suggest associations with different suitable habitats and serve as a basis for future models evaluating the ecological niches of different vector species. Understanding the temporal and spatial heterogeneity of triatomines in the southern United States will improve targeted interventions of vector control and will guide public outreach and education to reduce human and animal contact with vectors and reduce the risk of exposure to *T. cruzi*.

## INTRODUCTION

Triatomine insects (Reduviidae: Triatomine) are vectors of *Trypanosoma cruzi*, the protozoan parasite responsible for Chagas disease in over 5.7 million people throughout the Americas.^1^ Triatomines are obligatory hematophagous arthropods and become infected with *T. cruzi* when blood feeding on infected mammalian hosts. The parasite replicates in the gut of the insect, and the insect passes the parasite through fecal material. The study of these vectors has been key to public health initiatives aimed at reducing risk of Chagas disease.

Eleven species of triatomine insects have been recorded across the southern United States, where they are colloquially known as “kissing bugs” or “conenose bugs.”^2^ With seven species, Texas has the greatest triatomine species richness of any state: it is home to the western limit of *Triatoma sanguisuga* (“eastern conenose bug”), the eastern limit of *Triatoma protracta* (“western conenose bug”), and the northern limit of *Triatoma gerstaeckeri*.^2,3^ In contrast to areas of Central and South America, where domestic and peridomestic populations of triatomines exist, the species in the United States are almost exclusively sylvatic.^2,4^ Nymphs are rarely documented in human domiciles,^5^ and the triatomines found in peridomestic and domestic settings are understood to be mainly dispersing adults, likely driven by mate and/or blood meal–seeking behaviors.^6–9^ Although human contact with such dispersing adults poses potential risk of spillover transmission of *T. cruzi* to humans, studies of triatomine dispersal phenology in the United States are limited.^5,7,10,11^

Historical records of triatomines show species distributed across 29 United States states^2,12^; however, occurrence maps at the scale of county and state levels do not reflect the complexity of unique species distributions and are poor indicators of the spatial risk of exposure.^13^ This is particularly true in areas where there is high species diversity, such as Texas.^2,14^ Species distribution modeling efforts of triatomines in the United States are frequently limited by small sample sizes from broad geographic areas.^15,16^ Because infection prevalence^17^ and host associations^18^ are known to vary among triatomine species, risk of parasite transmission to humans and domestic animals likely varies depending on the triatomines found in a local area. A more precise understanding of occurrences of individual triatomine species is needed as a basis for robust studies analyzing habitat suitability and potential for range expansion due to climate change. The objective of this study was to determine temporal and spatial variation of triatomine activity and define contemporary and specific occurrences of *Triatoma* spp. across Texas.

## MATERIALS AND METHODS

### Specimen collection.

From May 2012 to November 2016, we acquired triatomines using standard entomological trapping techniques and a citizen science program as previously reported.^17^ The collection techniques used by members of our research group included black lights and mercury vapor lights, dry ice–baited white sheets, manual searching with lights at night, and active searching/destructive sampling of woodrat (*Neotoma* spp.) nests and other nidicolous habitats as described by others.^19–22^ In addition, stand-alone traps were set for overnight captures, including a standard universal black light trap (Product #2851A; BioQuip Products, Rancho Dominguez, CA) and the MegaCatch ULTRA Mosquito Trap (EnviroSafe Technologies International Limited, Albany, New Zealand). The MegaCatch ULTRA included an octenol-based attractant. Additional captures were attempted using yeast-baited traps consisting of baker’s yeast in a water chamber set on a sticky trap and partially covered with an inverted container, similar to descriptions from previous studies.^23,24^

### Laboratory processing.

Adult triatomines were morphologically identified to species,^3^ sexed, and measured. To mitigate risk of exogenous DNA contamination from the external exoskeleton in downstream processing, triatomines were soaked in 50% bleach for 15 seconds and then rinsed in distilled water.^25^ Hindguts were dissected using sterile forceps and dissecting scissors. Upon dissection, the relative amount of blood was scored (1 = no blood, desiccated guts; 2 = no blood, guts visible; 3 = traces of blood in gut; 4 = blood present, but either not much or not fresh [dried]; 5 = large amount of fresh blood) in a similar way to previous publications,^20,26,27^ but by direct observation during dissection and with the addition of a category (“1”) that considered that many of the samples we received had been dead for an undetermined time before submission through the citizen science program.

Some specimens were unable to be identified using morphological characteristics, which included nymphs, incomplete or damaged specimens, and those with aberrant morphological characteristics. A subset of these specimens, along with representative morphologically identified specimens, was subjected to molecular determination of species. DNA from leg or hindgut tissue was extracted using the Omega E.Z.N.A Tissue DNA kit (Omega Bio-Tek, Norcross, GA), and polymerase chain reaction (PCR) amplification of the mitochondrial cytochrome *b* gene was done.^28^ Cytochrome *b* PCR reactions consisted of 1 μL DNA, 0.6 μL of each 10 μM primer, and FailSafe PCR Enzyme Mix in PreMix E (Epicentre, Madison, WI) in a total volume of 15 μL. PCR amplicons were visualized on a 1.5% agarose gel stained with ethidium bromide. Target amplicons were purified using ExoSAP-IT (Affymetrix, Santa Clara, CA) and bidirectionally sequenced (Eton Bioscience, Inc., San Diego, CA). Sequences were visually inspected for quality in Geneious version 8 (http://www.geneious.com)^29^; unknown species identifications were inferred based on alignment with specimens of known species in a boot-strap consensus phylogentic tree using 1,000 replicates and the neighbor-joining method in MEGA7.^30^ Sequences were deposited to Genbank (Accession Numbers KY305689–KY305710; KY305712–KY305737).

### Temporal and spatial analyses.

Cross-correlation functions were used to estimate the correlation between the weeks of capture for the most frequently collected species (*T. gerstaeckeri*, *T. sanguisuga*, *Triatoma indictiva*, *Triatoma lecticularia*, and *Triatoma rubida*) with known capture weeks. The analysis was restricted to the adult triatomines captured alive by citizen scientists, which are representative of dispersing adults.^6–9^

Locations of citizen-submitted specimens were geocoded; locations were vetted for accuracy and precision using Google Earth version 7 (http://www.google.com/earth/). ArcMap 10.1 (ESRI, Redlands, CA) was used to create maps of locations where *Triatoma* spp. were encountered.

Spatial point pattern analysis was completed in R^31^ for the five most frequently collected *Triatoma* species. The Kelsall and Diggle method^32,33^ was used to determine locations of significant occurrence for individual *Triatoma* species by comparing expected density ratios of each “species of interest” (i.e., *T. gerstaeckeri*, *T. sanguisuga*, *T. indictiva*, *T. lecticularia*, or *T. rubida*) to the “noninterest *Triatoma* species” (i.e., the other four *Triatoma* spp. that served as a comparison to the species of interest for the analysis) (see R code in Supplemental Material). Kernel density estimates for collection locations of the species of interest were calculated along a two-dimensional grid within Texas. Kernel density estimates for the collection locations of noninterest species were calculated along the same grid, and the ratio of the species of interest and noninterest species estimates was calculated at each grid point. The natural log of the ratio at each point was used as the “observed data set.” A permutation test was used to assess significance of the observed data set. The collection locations of all species were randomly assigned to “species of interest” or “non-species of interest” categories, and kernel densities, ratios, and natural logs were computed as mentioned earlier. This was repeated for 100 simulations, and the observed dataset for the species of interest was compared with the permutated datasets. Observed statistics greater than 99.5% of those statistics derived from permutated data were classified as significant, indicating higher than expected observations of the species of interest compared with the noninterest species. Observed data set calculations lower than 0.5% were also classified as significant, indicating lower than expected observations of the species of interest.

## RESULTS

### Specimen collection.

From May 2012 to November 2016, we obtained 3,215 triatomine specimens. The majority (89.7%; 2,883 specimens) were obtained through citizen collections; this included a subset of the 1,980 specimens from the years 2012 to 2014 that were included in a previous publication highlighting the citizen science approach to vector collections.^17^ In addition to citizen-submitted specimens, we collected 332 triatomines (10.3%) in the field using multiple collection techniques. Specimens came from more than 534 unique locations, although some triatomines submitted by citizens did not include exact locations. Most of the specimens were from Texas (93.5%; 3,006 specimens), but additional samples were submitted from 17 other states (Table 1). All of the triatomines collected by members of our research team occurred in Texas; citizen scientists collected all of the non-Texas samples. There were 2,334 triatomines collected by citizen scientists for which exact capture locations were specified (Table 2). Triatomines were frequently collected from peridomestic areas (29.1%), dog kennels (37.8%), and indoors (26.0%) (Table 2). Of the 46 nymphs collected indoors, in all but seven cases, the nymph found was a solitary, late-stage (instar 4 or 5) nymph. The seven exceptional cases included three encounters of two (late stage) nymphs each, one encounter of two (one early stage and one late stage) nymphs, one encounter of four nymphs (two early stage and two late stage) suspected to have been feeding on a cat under a bed, one encounter of five nymphs (three early stage and two late stage) in a poorly sealed house in a very rural area, and one encounter of five (early stage; instar 1–3) nymphs found in a home after the submitter had been bitten multiple times in bed. In the cases in which a triatomine was found in a bedroom, our research team facilitated contact between the citizen and the state health department.

**Table 1:** Triatomine specimens collected from multiple states, 2012–2016

*Triatoma* species	State of vector collection																		
	AL	AZ	CA	FL	GA	IN	KS	KY	LA	MO	NM	NC	OH	OK	SC	TN	TX	VA	Total
*Triatoma gerstaeckeri*	–	–	–	–	–	–	–	–	–	–	–	–	–	–	–	–	2,045	–	2,045
*Triatoma indictiva*	–	–	–	–	–	–	–	–	–	–	–	–	–	–	–	–	132	–	132
*Triatoma lecticularia*	–	–	–	–	–	–	–	–	–	–	–	–	–	–	–	–	110	–	110
*Triatoma neotomae*	–	–	–	–	–	–	–	–	–	–	–	–	–	–	–	–	3	–	3
*Triatoma protracta*	–	14	6	–	–	–	–	–	–	–	1	–	–	–	–	–	10	–	31
*Triatoma rubida*	–	87	–	–	–	–	–	–	–	–	7	–	–	–	–	–	110	–	204
*Triatoma sanguisuga*	4	–	–	56	1	2	1	1	5	2	–	1	1	2	–	2	421	7	506
Unknown (adult)*	–	–	–	–	–	–	–	–	–	–	–	–	–	–	1	–	54	–	55
Unknown (nymph)†	–	3	–	–	–	–	–	–	–	–	2	–	–	1	–	1	121	1	129
Total	4	104	6	56	1	2	1	1	5	2	10	1	1	3	1	3	3,006	8	3,215

*Triatoma* spp. were identified using morphological characteristics.

* Specimen morphological identification was not possible (missing key morphological features).

† No morphological key for nymphs exists.

**Table 2:** Locations of triatomine collections by citizen scientists

	Location	*Triatoma* species										
		*T. gerstaeckeri*	*T. indictiva*	*T. lecticularia*	*T. neotomae*	*T. protracta*	*T. rubida*	*T. sanguisuga*	Unknown (adult)	Unknown (nymph)	Total	Percent of total*
Indoors	Primary residences†	159	20	29	–	20	97	138	14	45	522	22.4
	Non-residences‡	9	–	2	–	–	–	8	–	–	19	0.8
	Hunting cabins	8	–	1	–	–	–	56	–	1	66	2.8
	*Indoors total*	*176*	*20*	*32*	*–*	*20*	*97*	*202*	*14*	*46*	*607*	*26.0*
Outdoors	Dog kennel§	567	24	15	–	–	–	38	13	21	678	29.1
	Peridomestic environment¶	605	58	28	–	2	26	132	13	17	881	37.8
	Barns and chicken coops	12	6	23	–	–	–	10	1	2	54	2.3
	Outdoor-other‖	23	2	1	–	–	–	14	1	2	43	1.8
	Outdoors, exact location not specified	46	–	3	–	1	–	18	–	3	71	3.0
	*Outdoors total*	*1,253*	*90*	*70*	*–*	*3*	*26*	*212*	*28*	*45*	*1,727*	*74.0*
*Not specified*		*356*	*10*	*7*	*1*	*1*	*81*	*67*	*5*	*21*	*549*	
Total		1,785	120	109	1	24	204	481	47	112	2,883	–

When known, collection sites of triatomines were classified as “indoors” or “outdoors,” with subcategories. This table includes all triatomines (alive and dead) collected by citizen scientists.

* Percent is calculated from the subtotal (2,334) of triatomines for which exact location was specified by collector.

† Human residences with continuous, regular occupancy.

‡ Office and clinic buildings.

§ These were primarily multi-dog, outdoor, open air kennels.

¶ Including porches, patios, garages, and outer surfaces of houses.

‖ Triatomines collected from a deer hunting stand (one nymph), in a car (one adult, unknown species), near a woodrat nest (two *T. gerstaeckeri* and one nymph), parking lots (one *T. gerstaeckeri* and one *T. sanguisuga*), swimming pools or water buckets (all dead; 10 *T. gerstaeckeri*, two *T. indictiva*, one *T. sanguisuga*), camping tents (five *T. gerstaeckeri*), near blacklights situated 10–20 m high on a tower (Lee County, TX; five *T. gerstaeckeri*, one *T. lecticularia*, and 11 *T. sanguisuga*), and by an individual while trapping raccoons during the night (one *T. sanguisuga*).

Of the 332 specimens collected by our team, 235 were alive at the time of capture and categorized by the method of search (Supplemental Table 1). Despite attempts over multiple nights in different areas using stand-alone collection traps with black and/or mercury vapor light, carbon dioxide attractants, and synthetic pheromone attractants, no triatomines were captured in such unattended devices. At one south Texas site in June 2013, a team of six people collected 86 *T. gerstaeckeri* and two *Triatoma neotomae* in a matter of hours; these collections were made in a peridomestic area under a high, bright, security-type light, in an open area surrounded by dense brushy vegetation. In addition, there were two instances of *T. sanguisuga* flying and landing on a collector, as well as one *T. protracta* discovered on the vest of a collector at night. Four triatomines were collected during early morning hours from the outer wall of the tent we camped in during fieldwork. Additional collection details can be seen in the Supplemental Material (Supplemental Table 1).

### Characterization of specimens.

Triatomines collected were predominantly *T. gerstaeckeri* (63.6%), with *T. gerstaeckeri* and three other species—*T. indictiva*, *T. lecticularia*, *T. sanguisuga*, and *T. rubida*—comprising 93.7% of all specimens collected in Texas (Table 1). For those specimens for which sex was able to be determined (Supplemental Table 2), the proportion of females collected was greater than males collected for all species except *T. lecticularia*, *T. protracta*, and *T. neotomae* (small sample size). Sexes of nymphs were not determined. *Triatoma gerstaeckeri* was the largest size species included in the collection (Supplemental Table 3). Specimens measuring outside of the range of expected lengths were rare.^3^ A total of 1,459 live-captured triatomines were scored for blood meal (Supplemental Table 4); 50.6% were starved (blood meal scores of 1–2), whereas 29.6% had evidence of a recent blood meal (scores of 4–5). Triatomines captured in houses were more likely to be starved (64.6%; 95% confidence interval [CI] 58.2–70.5%) than triatomines collected from dog kennels (30.2%, 95% CI 25.3–35.5%) (Supplemental Table 4).

Morphological identification of 48 specimens was confirmed by amplification and sequencing of the cytochrome *b* gene (Supplemental Figure 1). For the subset of molecularly tested triatomines for which the species identification was assigned based on morphologic features, all species identifications that were determined based on DNA sequence analysis confirmed the same identity. The consensus tree revealed distinct clades for all included species, except for *T. sanguisuga* and *T. indictiva*, which were indistinguishable based on this genetic region.

### Temporal patterns.

**Figure 1.**
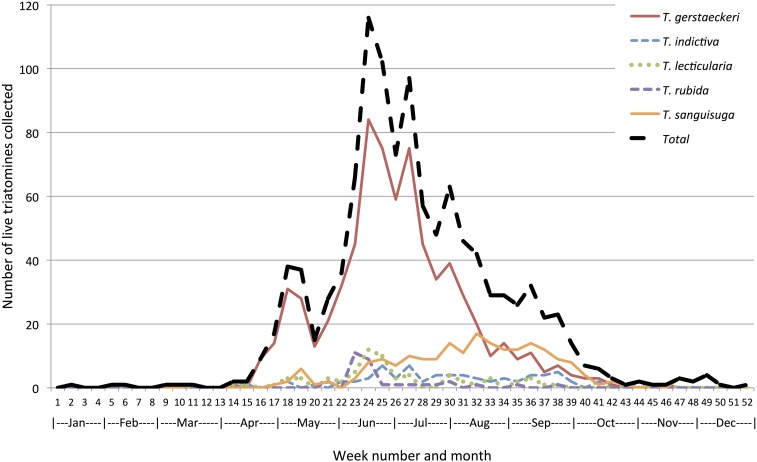
Phenology of collection of live triatomines of five species. Seasonal occurrence of five species of triatomines (and the total of those five species) collected alive by citizen scientists in Texas, 2012–2016. Of 39 specimens collected from November through March, 36 had locations specified, of which 27 (75.0%) were found indoors. In addition, four were found outdoors near animal quarters, including three in dog kennels and one in a chicken coop. This figure appears in color at www.ajtmh.org.

**Figure 2.**
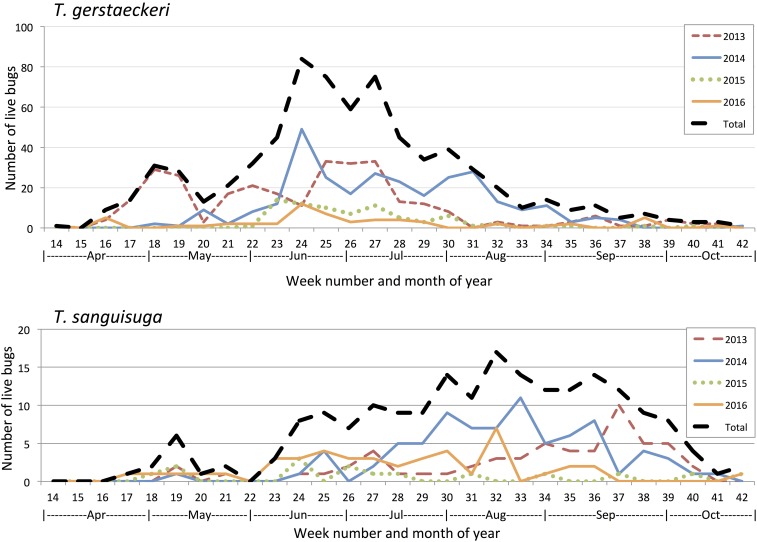
Yearly phenology of collection of live triatomine species from Texas. Citizen scientists in Texas opportunistically collected live *Triatoma gerstaeckeri* and *Triatoma sanguisuga*, 2013–2016. This figure appears in color at www.ajtmh.org.

Triatomines were mainly collected from April through October (Figure 1). Collections of *T. gerstaeckeri* were mainly from late April to late September, peaking in June/July, whereas collections of *T. sanguisuga* were mainly from mid-June to early October, peaking from August–September (Figure 2). The cross-correlation function revealed significant correlation in the temporal capture of live triatomines of different species by citizen scientists, which varied by year (Supplemental Table 5). In 2013, *T. gerstaeckeri* had a peak correlation 11 weeks before *T. sanguisuga* and *T. gerstaeckeri* peak activity was indistinguishable from peak activities of *T. indictiva* and *T. lecticularia*. In 2014, *T. gerstaeckeri* had a peak correlation 6 weeks before *T. sanguisuga* and *T. indictiva*, and at 3 weeks before *T. lecticularia*. In 2015, *T. gerstaeckeri* had a peak correlation one week before *T. sanguisuga*, 2 weeks before *T. indictiva*, and there was no significant correlation with *T. lecticularia*. In 2016, *T. gerstaeckeri* had a peak 8 weeks before *T. sanguisuga*, although with a second smaller peak approximately 1 week prior until 1 week after peak *T. sanguisuga* captures. In 2016, the *T. gerstaeckeri* peak was indistinguishable from *T. lecticularia* peak and was one week after the *T. rubida* peak.

Across the 3.5-year study period, only 39 specimens were collected in the winter months from November through March in Texas. Of 36 with specified capture locations, 27 (75.0%) were found indoors. In addition, four were found outdoors near animal quarters, including three in dog kennels and one in a chicken coop; three triatomines were collected from peridomestic areas; and one triatomine was found in a parking lot. Fifteen (38.5%, 95% CI 24.9–54.1%) of the live specimens found during the winter were nymphs. In comparison, of the 1,210 live triatomines found from May through October, only 31 (2.6%, 95% CI 1.8–3.6%) were nymphs.

### Geographic distribution and spatial analyses.

Specimens were collected from 123 counties in Texas and from Arizona, California, Florida, Georgia, Indiana, Kansas, Kentucky, Louisiana, Mississippi, Missouri, New Mexico, North Carolina, Ohio, Oklahoma, South Carolina, Tennessee, and Virginia (Supplemental Figures 2 and 3). Precise capture locations allowed mapping of *T. gerstaeckeri* (*N* = 1,906), *T. indictiva* (*N* = 128), *T. lecticularia* (*N* = 105), *T. neotomae* (*N* = 3), *T. protracta* (*N* = 10), *T. rubida* (*N* = 107), and *T. sanguisuga* (*N* = 377) in Texas (Supplemental Figure 3).

**Figure 3.**
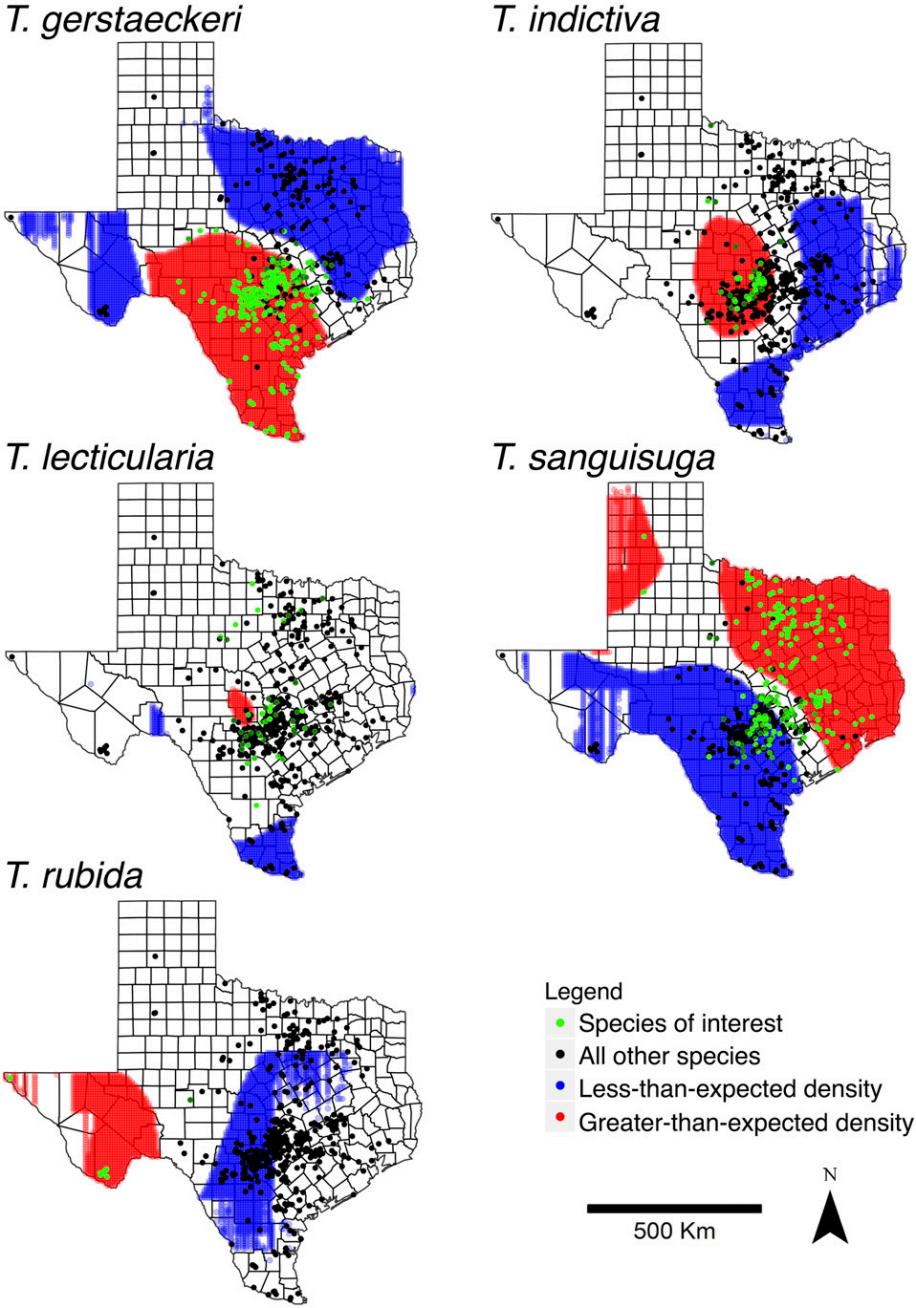
Maps indicating areas of relatively unique distributions of five triatomine species in Texas. Higher than expected densities of *Triatoma* spp. are highlighted in red and lower than expected densities are highlighted in blue. This figure appears in color at www.ajtmh.org.

We used the Kelsall and Diggle method to compare the density of each triatomine species to all the other species (“noninterest species”) in Texas (see R code in Supplemental Material). The spatial analysis was performed for *T. gerstaeckeri* (*N* = 1,906), *T. indictiva* (*N* = 128), *T. lecticularia* (*N* = 105), *T. rubida* (*N* = 107), and *T. sanguisuga* (*N* = 377). The resulting maps (Figure 3) indicate areas of relative higher encounter rates in west Texas for *T. gerstaeckeri*, in central Texas for *T. indictiva*, in central to northwestern Texas for *T. lecticularia*, in west Texas for *T. rubida*, and in north and east Texas for *T. sanguisuga*.

## DISCUSSION

Although Chagas disease is increasingly recognized as a disease of human and veterinary significance in the southern United States, there have been relatively few studies of the phenology and spatial patterns of triatomine vectors in the United States. We established a collection of 3,215 triatomines, mainly from Texas, to examine unique phenological and spatial patterns.

Although the majority (89.7%) of specimens were collected by citizen scientists, our research team collected 235 live specimens using a variety of methods with varying success (Supplemental Table 1). As others have found,^14^ no triatomines were captured in unattended devices. Wood reported collecting an average of 2.6 bugs per hour when searching woodrat nests and 11.6 bugs per hour when collecting strategically from a road overcrossing where a *Triatoma* aggregation was reported.^34^ Similarly, when we searched nidiculous habitats, we found approximately 3.8 bugs per hour, which was in contrast to collecting 88 bugs in a few hours during one serendipitously timed field sampling in south Texas. Our findings add to the evidence that collection of triatomines from sylvatic environments is a time-intensive undertaking.

The five most commonly encountered species—*T. gerstaeckeri*, *T. indictiva*, *T. lecticularia*, *T. rubida*, and *T. sanguisuga*—are consistent with species encountered in other studies in Texas.^5,14,35,36^ The preponderance of *T. gerstaeckeri* may not necessarily reflect a higher relative abundance of this species in nature, since citizen-collected triatomines were frequently encountered in peridomestic habitats, where adult *T. gerstaeckeri* are known to occur.^5^ As noted by others,^37^ the relatively low burden of human Chagas disease in the United States is likely partially due to infrequent vector colonization of human residences. Most of the triatomine species found in the United States occur mainly in the sylvatic environment and are rarely reported as having established populations in domestic settings.^5^ However, many of the triatomines in the current study (92.9%; Table 2) were encountered indoors, in peridomestic settings, or in dog kennels and represent a risk of spillover transmission of *T. cruzi* to humans and domestic animals. Therefore, although triatomine egg laying, nymphal development, and infection with *T. cruzi* may mainly occur in the sylvatic environment in the United States,^38^ dispersing adult triatomines found in the domestic or peridomestic setting are an important risk to human/animal health.^5,39^ Although *T. gerstaeckeri* are frequently encountered in peridomestic settings, rare findings of nymphs (as others have found)^40^ suggest that these triatomines are dispersing adults, rather than species with established lifecycles in the domestic setting. However, it is also possible that adult triatomines are more easily noticed and submitted by citizen scientists than smaller, less conspicuous nymphs. For example, *T. sanguisuga* nymphs have been described as “especially shy in the nymphal stages.”^11^ Rigorous field studies of host associations are needed to understand the sylvatic sources of triatomines in the United States.

We found a clear sex bias in submitted insects, with more females collected across all species, except *T. lecticularia* and *T. protracta* (Supplemental Table 2). Differences in behavior by sex in triatomines found in the United States have not been well studied, and few prior studies of these species document sex ratios of collections. In two studies, *T. rubida* females were likely to defecate during a blood meal, although males were not,^41,42^ which would affect risk of *T. cruzi* transmission to the host. Although no studies of effect of sex on flight dispersal of triatomine species have been conducted in the United States, studies of *Triatoma infestans* and *Rhodnius pallescens* in South America found sex-based differences in likelihood of flying, distance flown, and speed of flight.^43–46^ Studies of *T. gerstaeckeri*, *T. protracta*, and *T. sanguisuga* in the United Studies,^6,11,47^ and studies of *T. gerstaeckeri*, *T. lecticularia*, and *T. rubida* in Mexico,^40,48^ have found a range of female to male ratios in a variety of settings. Sex-biased survivorship is one additional mechanism that could have yielded more females in the present study, but this has been poorly studied.^11^

We found distinctive periods of peak activity, with *T. gerstaeckeri* most commonly collected from April to September, and *T. sanguisuga* was more active during June–October. In fact, based on cross-correlation analyses of 2013–2014 and 2016 data, *T. gerstaeckeri* captures peaked 6–11 weeks before *T. sanguisuga*. This is in agreement with previous findings of activity from May to August.^5,11^ In this study, adult triatomine specimens collected in the domestic/peridomestic habitat and noted as alive at the time of collection were considered to likely be dispersing.^6,9^ Low nutritional status, lack of nearby blood meal sources, anthropogenic changes, and temperature have been proposed as the main drivers of flight dispersal for *T. protracta*,^6,34^ although similar studies have not yet been done for other species found in the United States. We have observed copulation of dispersing adult *T. gerstaeckeri* in April–June collections in southern Texas (unpublished data), suggesting that mating is another potential driver of dispersal behavior. Others have found that dispersing adults attracted to traps are more likely to be starved,^6,11^ suggesting that need for a blood meal is a main driver of flight dispersal of adult triatomines in the United States. Only 29.6% of specimens collected had evidence of recent blood meals, whereas 50.6% had no or little evidence of blood in the gut (Supplemental Table 4), which suggests that active, exposed (and therefore likely to be captured) triatomines are likely searching for a blood meal. Notable exceptions were triatomines found in barns/chicken coops, wildlife nests/dens, and dog kennels, which more commonly had a recent blood meal (Supplemental Table 4). Triatomines in these settings may have been found after feeding on the available animals. In particular, dogs are a known blood meal source for triatomines in kennels^49^ and triatomines have well-described associations with chickens throughout the Americas.^48,50^

Relative to the more northern distribution of *T. sanguisuga* in Texas, it might also be possible that the more southern distribution of *T. gerstaeckeri* would have earlier warmer temperatures that would induce flight dispersal.^6^ Prior phenology studies found that *T. rubida* and *T. gerstaeckeri* primarily overwinter as fifth instar nymphs, whereas *T. sanguisuga* overwinters as a fourth instar nymph.^11,19^ In addition, one study found *T. rubida* males molted to dispersing adults earlier than females.^19^ Studies of the *Neotoma* spp. rodent/*T. protracta* relationship led Sjogren and Ryckman to hypothesize that death of a blood source would result in nutritional deprivation and subsequent dispersal of triatomines. Relative to the well-studied *Neotoma*/*T. protracta* relationship, little is known about the host preferences of *T. gerstaeckeri* and *T. sanguisuga*^3,38^; perhaps unique host associations with different natural histories result in distinct dispersal activity.

When comparing the phenology and total submissions across the three years of the study, we noted an overall lower number of triatomine submissions in 2015 compared with the previous two seasons (Figure 2). Although the passive sampling technique of citizen science submissions does not allow for robust conclusions regarding population dynamics, the standardized collection efforts by members of our research team also yielded fewer specimens in 2015 relative to the two prior years. It is also interesting to note that in 2015 the cross-correlation analysis found the peak captures of *T. gerstaeckeri* to be only one week before *T. sanguisuga*, when in the other study years, *T. gerstaeckeri* captures peaked 6–11 weeks before *T. sanguisuga*. It may be possible that climate conditions affected triatomine populations: Texas suffered a severe drought in 2011,^51^ and precipitation gradually increased over the next few years, until it returned to average conditions in 2016.^52^ Studies in other disease systems have shown that temperature and precipitation can influence vector abundance and risk of disease transmission.^53,54^

The use of molecular methods for triatomine species determination is valuable, particularly for low-quality specimens and nymphs. The sequences generated using the mitochondrial cytochrome *b* target were able to differentiate all species in Texas, except for *T. sanguisuga* and *T. indictiva*. The genetic similarity of these two species is consistent with a previous report examining the same genetic region.^20^ There has been conflicting classification of *T. indictiva*, with some including it as a subspecies of *T. sanguisuga*^55^ and others separating it as its own species.^3^ A previous study of *T. sanguisuga* collected from a focal location in Louisiana revealed distinct groups of the triatomine species based on two genetic regions.^56^ In this study, individuals morphologically identified as *T. indictiva* were found in a more focal region than *T. sanguisuga* (Figure 3), although *T. indictiva* has previously been recorded throughout additional southwestern states.^3^

Our investigation of spatial distribution revealed heterogeneities in the collection of different triatomine species across Texas. Mapping the precise collection localities was possible for many specimens of the five species most frequently collected: *T. gerstaeckeri*, *T. sanguisuga*, *T. indictiva*, *T. lecticularia*, and *T. rubida*. The data on frequency of encountering these species are epidemiologically meaningful, since vectors must be present for vector-borne disease risk to humans and domestic animals. Given that *T. cruzi* infection prevalence varies across species,^17^ understanding species distributions can aid in building risk models. Point pattern analyses were specifically used to compare the collection localities of the five most frequently collected species. Each individual species of interest was compared with the collective locations of the four other species, revealing locations where the species of interest was more likely to be encountered than the other four species. Although the unique occurrences were expected in north/east Texas for *T. sanguisuga* (the “eastern cone nose bug”) and in west Texas for *T. rubida*, we also identified relatively high reports of *T. lecticularia* and *T. indictiva* in central Texas, as well *T. gerstaeckeri* in south and west Texas. Our data advance previous work mapping historical and other contemporary collections that were based on small sample sizes and/or restricted to county-level spatial scale,^5,14^ and could be used to improve the specificity of models attempting to delineate species distributions and potential expansions.^15,16^ A limitation of the dataset is non-uniform citizen science sampling, and several regions of Texas had very few submissions of triatomines, although triatomines are known to occur in those regions^14,36^ and despite many submissions of other insects and similar-looking species, such as “wheel bugs” (*Arilus cristatus*) and “leaf-footed bugs” (*Leptoglossus* spp.).^17^ The areas with fewer submissions in west and south Texas are likely due to low human population densities and/or lack of outreach to those areas. Further investigations of triatomine habitat suitability modeling and *T. cruzi* infection status would allow greater ability to assess risk of disease transmission to humans and animals.

This study demonstrates the widespread occurrence of triatomines in Texas and other southern states, with adult dispersal occurring during the summer months and the greatest number of specimens collected from central Texas. These findings can be used to guide public health interventions aiming to reduce risk of *T. cruzi* transmission to humans and domestic animals.

## Supplementary Material

Supplemental Figure.
