# Supplementary material for: Bionomics and Spatial Distribution of Triatomine Vectors of Trypanosoma cruzi in Texas and Other Southern States, USA
Source: Am J Trop Med Hyg. 2017 Nov 6;98(1):113–21. doi: 10.4269/ajtmh.17-0526 (PMC5928729; doi:10.4269/ajtmh.17-0526)
Supplement: Supplementary file 1 [file tpmd170526.SD1.pdf]

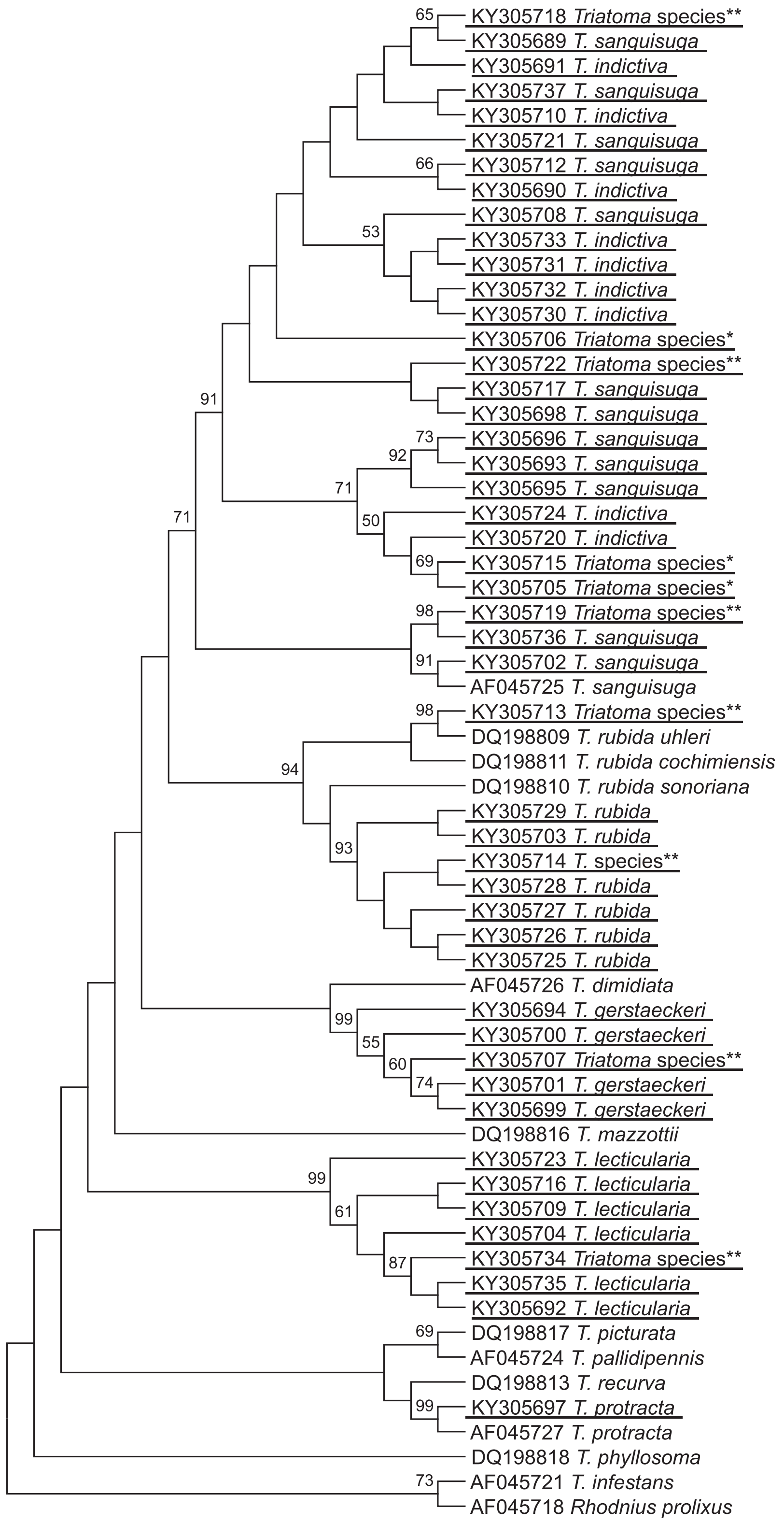

Supplemental Figure 1. Evolutionary relationships among *cytochrome b* sequences. Sequences from field samples (underlined) and existing sequences (not underlined) were used to construct a phylogenetic tree. For field samples, the morphologically determined identification is listed; samples unable to be identified using morphological features are noted with an asterisk (*adult specimen, **nymph specimen). The evolutionary history was inferred using the Neighbor-Joining method, and the optimal tree is shown. The percentage of replicate trees in which the associated taxa clustered together in the bootstrap test (1,000 replicates) is shown next to the branches; only those with values of greater than 50% are displayed. Evolutionary distances were computed using the Tamura-Nei method and are in the units of the number of base substitutions per site. The analysis involved 61 nucleotide sequences. All ambiguous positions were removed for each sequence pair. There were a total of 197 positions in the final dataset. Evolutionary analyses were conducted in MEGA5 (Tamura et al., 2011).

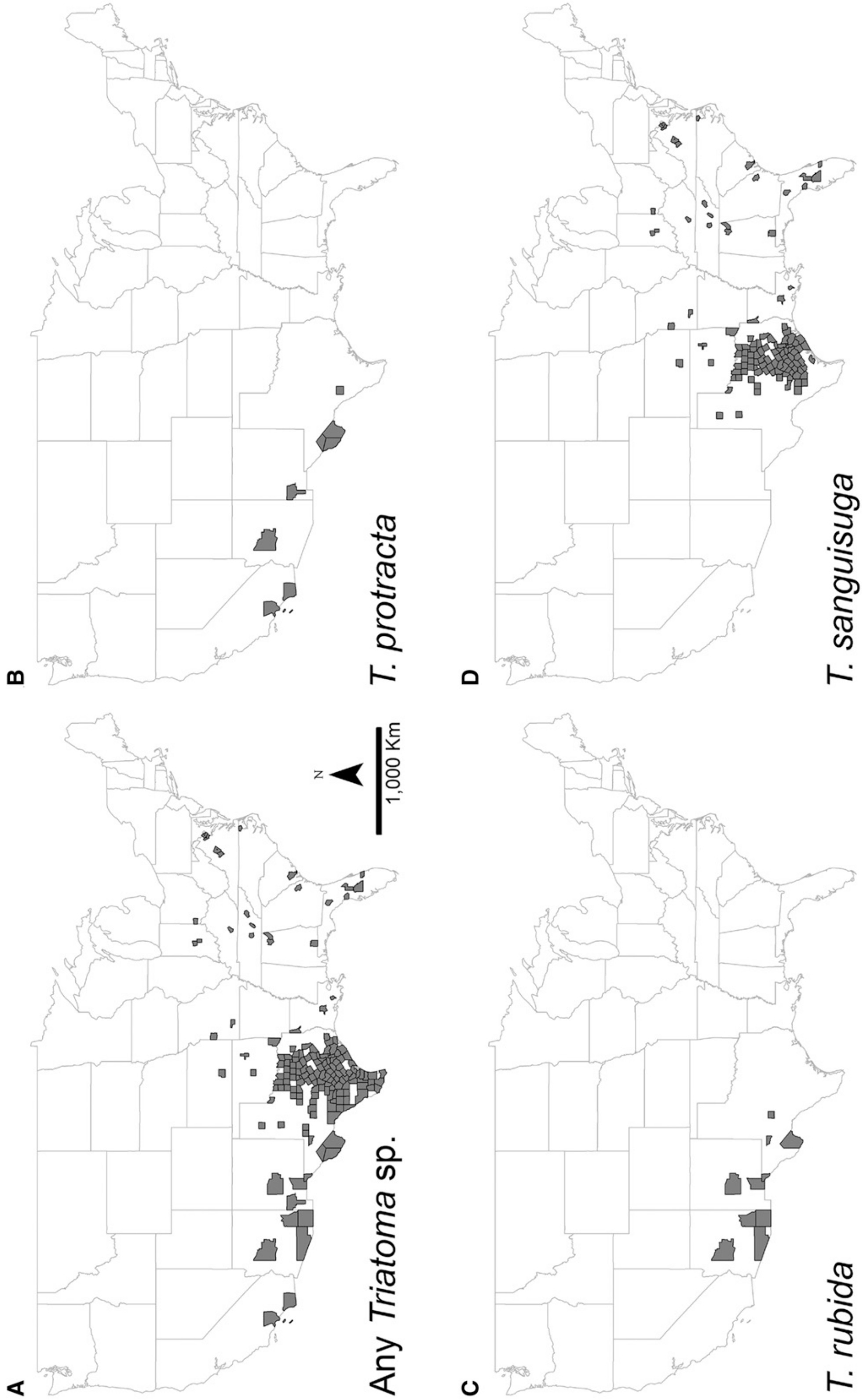

Supplemental Figure 2. Geographic distribution of triatomine collections across the United States, with detailed maps of three species collected from multiple states, 2012–2016. (**A**) Specimens of any species (including *Triatoma gerstaeckeri*, *Triatoma indictiva*, *Triatoma lecticularia*, *Triatoma neotomae*, *Triatoma protracta*, *Triatoma rubida*, and *Triatoma sanguisuga*) were collected from shaded counties: 123 counties in Texas and at least one county in each of 17 other states. (**B**) *T. protracta* was collected from four southwestern states. (**C**) *T. rubida* was collected from three southwestern states. (**D**) *T. sanguisuga* was collected from 15 states, mainly in eastern states.

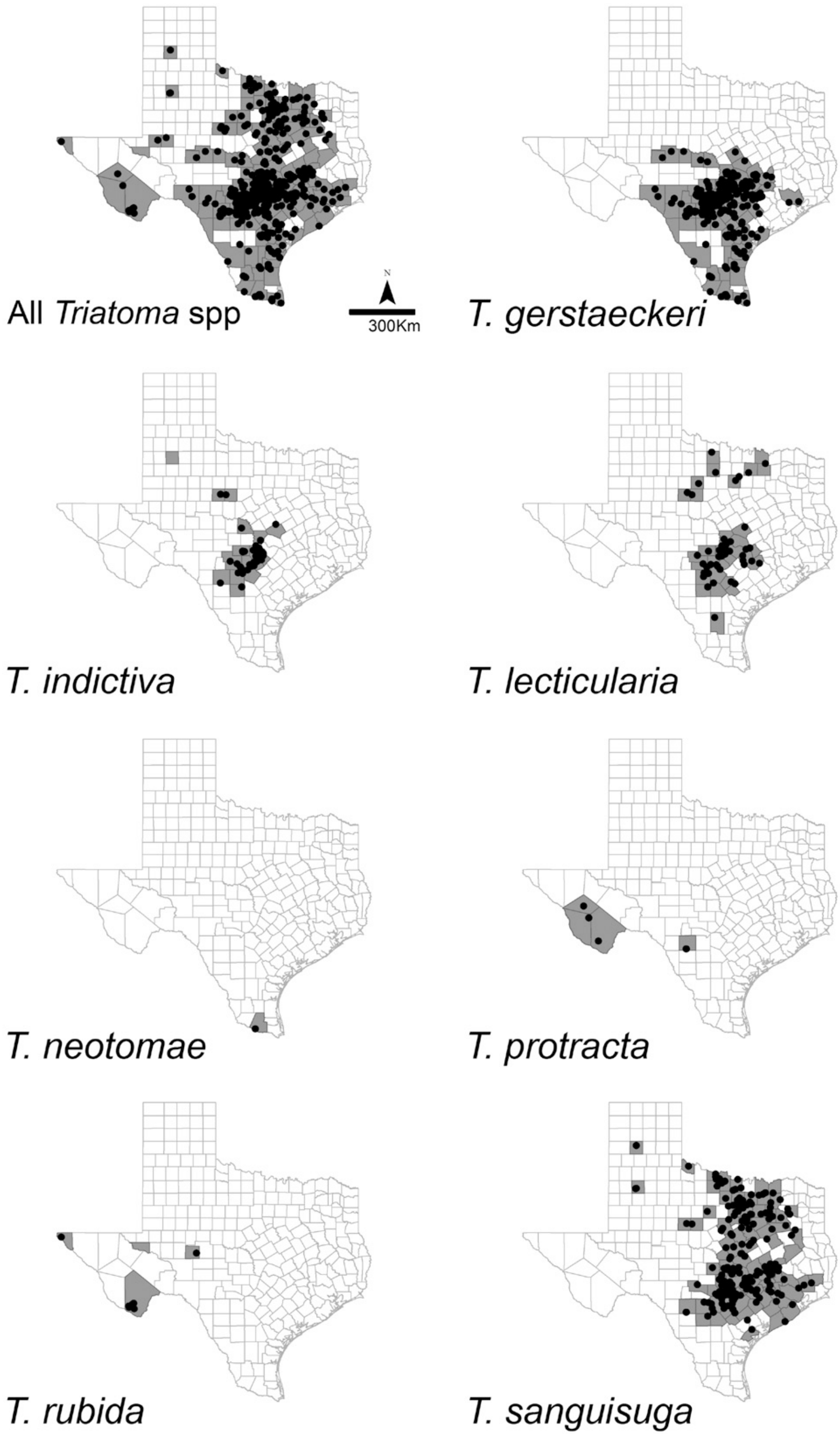

SUPPLEMENTAL FIGURE 3. Spatial distribution of triatomine collections in Texas, 2012–2016. Seven *Triatoma* species were collected in Texas. All counties represented by samples in the collection are shaded, and black points show those samples for which exact coordinates were known.

Supplemental Table 1

Methods of capture for live-caught triatomines captured by our research team

Method	Total triatomines	Estimated total effort (hours)	Estimated average triatomines/hour	Comments
Intentional				
Active searching around a lighted building, during night	122	44	2.8	Lighted walls can provide an excellent contrasting background to see triatomines, mainly useful during known activity seasons (summer)
Active/destructive searching of nidicolous habitats, during day*	17	4.5	3.8	Requires intense physical effort, but may result in more nymphs than other methods; likely success year-round
Active searching around a dog kennel, day and night	29	6.5	4.5	Multi-dog kennels may be more productive than individual kennels
Black light, carbon dioxide (dry ice), white sheet, occasional monitoring, during night†	5	5	1.0	Does not require constant presence at location, but unknown whether triatomines will leave location
Black light, carbon dioxide, white sheet, continuous monitoring, during night	7	4	1.75	Requires constant presence at trapping location, but human presence may increase attractive cues to vectors
Black light and white sheet, occasional monitoring, during night‡	1	2	0.5	Does not require constant presence at location, but unknown whether triatomines will leave location
Opportunistic				
Collected by laboratory members during field work, late night or early morning§	36	NA	NA	These captures were made incidental to nontriatomine-focused activities
Collected near dry ice on sheet in caves, set to attract soft ticks, during day	9	NA	NA	Dry ice was placed on a sheet laid on the ground; triatomines were captured approaching the dry ice
Collected from outer wall of tent, or in cabin, during late night or dawn¶	5	NA	NA	At dawn, triatomines can easily be seen walking on the outer surface of the tent by people in the tent
Other‖	4	NA	NA	

For intentional efforts, we estimated average triatomines captured per hour of effort; to account for spatial and temporal variation in our collection efforts, only events for which at least one triatomine was collected were included in the estimates. All triatomines listed here were captured from April to September. NA = not applicable.

* These were 13 nymphs and four adults collected from a woodpile and destructively sampled nests of *Neotoma* sp. in Uvalde County, Texas.

† Two *Triatoma* nymphs were attracted to a strategically-placed (where we suspected a rodent nest nearby) sheet laid on ground, lights (black lights and mercury vapor bulb), and dry ice as a source of carbon dioxide. Three adult *T. gerstaeckeri* were captured near vertically hung sheets.

‡ One adult *T. lecticularia* was found on a vertically hung sheet approximately one hour after we had set-up the light and sheet. After setting up, we had left the area, and neither dry ice nor human monitoring provided carbon dioxide or other chemical cues to attract this triatomine.

§ Triatomines found incidental to other field work activities, including 17 triatomines captured while sampling bats near caves or water sources, 10 triatomines captured from lights/sheets set to capture other insects (not primarily for triatomines), six triatomines captured while approaching laboratory members in the morning while checking small mammal traps, four triatomines captured while approaching laboratory members resting on porches, one triatomine captured in a dog shelter kennel, one triatomine found at dawn in closed Sherman trap (no mammal present), and one triatomine landed on a researcher and was found on her vest.

¶ Four adult *T. gerstaeckeri* were collected from the outer surfaces of the camping tent, after two people had spent approximately 5–6 hours in the tent. One adult *T. gerstaeckeri* was collected from inside a bunkhouse at a research facility.

‖ Three triatomines (all *T. gerstaeckeri*) found and captured while laboratory member was recreationally rock climbing. One *T. protracta* found in the restroom at a rest stop in Marfa, TX.

Supplemental Table 2

Sex distributions of triatomines

Species*	Females	%, 95% CI†	Males	%, 95% CI	Total
Triatoma gerstaeckeri	1,126	56.8% [54.6–58.9%]	857	43.2% [41.1–45.4%]	1,983
Triatoma sanguisuga	295	60.7% [56.2–64.9%]	191	39.3% [35.1–43.7%]	486
Triatoma indictiva	78	62.9% [54.1–70.9%]	46	37.1% [29.1–45.9%]	124
Triatoma lecticularia	50	47.2% [37.9–56.6%]	56	52.8% [43.4–62.1%]	106
Triatoma rubida	135	69.9% [63.1–76.0%]	58	30.1% [24.0–36.9%]	193
Triatoma protracta	15	48.4% [31.9–65.2%]	16	51.6% [34.8–68.0%]	31
Triatoma neotomae	–	–	2	100% [NA]	2
Total	1,699	58.1% [56.3–59.9%]	1,226	41.9% [40.1–43.7%]	2,925

Triatoma spp. proportions of males and females are shown. Percentages of females and males were calculated out of the total number of sexed individuals of each species collected. NA = not applicable.

* Some samples were not able to be sexed because of missing features. Those samples are not included in totals or calculations here. There were 39 *T. gerstaeckeri*, nine *T. sanguisuga*, six *T. indictiva*, one *T. lecticularia*, two *T. rubida*, one *T. neotomae*, and 128 *Triatoma* spp. nymphs. There were 55 *Triatoma* spp. adults of unknown species, of which 33 were females, 16 were males, and six were not able to be sexed.

† CI = confidence interval, calculated using Agresti–Coull method using the "binom" package in R (Project R, http://project-r.org).

Supplemental Table 3

Length distributions of triatomines

Species	Females		Males	
	No. measured	Mean length ± SD (mm)	No. measured	Mean length ± SD (mm)
Triatoma gerstaeckeri	955	27.7 ± 1.68	731	26.0 ± 1.62
Triatoma indictiva	77	21.3 ± 1.14	44	20.02 ± 1.15
Triatoma lecticularia	48	20.6 ± 1.35	46	19.3 ± 1.54
Triatoma neotomae	–	–	1	16
Triatoma protracta	13	17.3 ± 1.32	9	16.3 ± 1.33
Triatoma rubida	134	21.3 ± 1.31	57	18.3 ± 1.25
Triatoma sanguisuga	261	21.9 ± 1.85	166	20.4 ± 1.93

Triatoma spp. lengths in millimeters with standard deviations are shown. Not all specimens were measured because of missing body parts. The lengths of females included ovipositors. A total of 107 nymphs of various stages and species were measured, with a mean length of 15.2 ± 5.9 mm. SD = standard deviation.

Supplemental Table 4

Blood meal scores for live-captured triatomines

	Location	Bloodmeal score, % (n)					Total
		1	2	3	4	5	
Indoors	Primary residences	44.2% (100)	20.4% (46)	16.8% (38)	13.3% (30)	5.3% (12)	226
	Non-residences	44.4% (4)	33.3% (3)	22.2% (2)	-	-	9
	Hunting/fishing cabin	25.8% (8)	12.9% (4)	54.8% (17)	6.5% (2)	-	31
	Indoors total	*42.1% (112)*	*19.9% (53)*	*21.4% (57)*	*12.0% (32)*	*4.5% (12)*	*266*
Outdoors	Peridomestic environment	36.6% (222)	20.0% (121)	19.6% (119)	17.7% (107)	6.1% (37)	606
	Dog kennel	19.3% (59)	10.8% (33)	14.4% (44)	21.0% (64)	34.4% (105)	305
	Barn/chicken coop	10.0% (5)	6.0% (3)	14.0% (7)	22.0% (11)	48.0% (24)	50
	Outdoor-other	17.3% (18)	43.3% (45)	31.7% (33)	6.7% (7)	1.0% (1)	104
	Outdoors, exact location not specified	30.3% (10)	18.2% (6)	30.3% (10)	12.1% (4)	9.1% (3)	33
	Wildlife nest/den	11.1% (1)	22.2% (2)	22.2% (2)	11.1% (1)	33.3% (3)	9
	Outdoors total	*28.5% (315)*	*19.0% (210)*	*19.4% (215)*	*17.5% (194)*	*15.6% (173)*	*1107*
Not specified		34.9% (30)	20.9% (18)	19.8% (17)	14.0% (12)	10.5% (9)	86
	Overall total	**31.3% (457)**	**19.3% (281)**	**19.8% (289)**	**16.3% (238)**	**13.3% (194)**	**1459**

A total of 1,459 triatomines were collected alive by citizen scientists and our research team and scored for blood meal at time of dissection. Blood meal scores of 1–2 were considered "starved" and scores of 4–5 were considered as having a recent blood meal. Color highlighting indicates cells with higher percentages.

Supplemental Table 5

Cross-correlation functions analyses

		Lag in Weeks																						
	Species	-11	-10	-9	-8	-7	-6	-5	-4	-3	-2	-1	0	1	2	3	4	5	6	7	8	9	10	11
2013	*T. sanguisuga*	0.51	0.45	0.26	0.25	0.21	0.06	-0.23	-0.49	-0.43	-0.41	-0.42	-0.52	-0.51	-0.43	-0.28	-0.18	-0.15	-0.21	-0.15	0.01	0.12	0.12	0.16
	T. indictiva	-0.02	0.09	0.14	-0.02	-0.05	0.01	-0.31	-0.21	-0.04	0.34	0.63	0.72	0.16	0.06	-0.03	-0.08	-0.08	-0.05	-0.09	-0.08	0.06	-0.07	-0.09
	T. lecticularia	-0.22	-0.15	-0.07	-0.18	-0.16	-0.16	0.21	0.04	0.16	0.37	0.57	0.90	0.85	0.66	0.55	0.48	0.14	0.05	0.01	-0.04	-0.15	-0.11	-0.09
2014	*T. sanguisuga*	-0.06	-0.06	0.24	0.34	0.29	0.58	0.54	0.37	0.37	0.35	0.29	-0.11	0.05	-0.06	-0.20	-0.36	-0.31	-0.28	-0.22	-0.09	-0.15	-0.13	-0.12
	T. indictiva	-0.23	-0.03	-0.31	-0.08	0.16	0.58	0.29	0.28	0.17	0.17	0.00	0.06	0.08	0.05	-0.08	-0.22	-0.30	-0.36	-0.28	-0.07	0.04	0.08	0.04
	T. lecticularia	-0.21	-0.07	-0.39	-0.42	-0.11	0.26	-0.07	-0.01	0.79	0.30	0.01	0.60	0.53	-0.01	0.17	0.25	-0.32	-0.36	-0.15	-0.21	-0.16	-0.04	0.03
2015	*T. sanguisuga*	-0.16	-0.03	-0.04	0.00	-0.01	0.07	-0.02	-0.21	0.24	0.05	1.19	0.98	0.64	0.27	-0.03	0.41	-0.03	0.15	-0.09	-0.22	-0.05	-0.18	-0.06
	T. indictiva	-0.09	-0.07	-0.02	-0.08	0.00	-0.07	-0.08	0.03	-0.28	0.89	0.67	0.57	0.26	0.00	0.05	-0.04	0.13	-0.25	-0.16	-0.07	-0.16	-0.18	0.03
	T. lecticularia	0.14	-0.06	-0.24	0.29	0.22	0.34	-0.02	-0.22	-0.08	-0.41	-0.47	0.27	0.27	0.01	-0.10	-0.25	-0.01	-0.21	-0.02	-0.15	-0.28	-0.01	-0.06
2016	*T. sanguisuga*	-0.13	-0.26	-0.14	0.59	0.11	0.09	0.19	-0.02	0.04	0.20	0.41	0.35	0.44	0.06	-0.09	-0.22	-0.25	-0.09	-0.29	-0.05	-0.18	-0.05	-0.09
	T. lecticularia	-0.04	-0.10	0.11	0.21	0.06	-0.40	-0.36	-0.21	-0.17	-0.16	0.66	1.91	0.61	-0.08	-0.07	0.13	-0.09	-0.32	-0.13	-0.11	-0.06	-0.10	0.01
	T. rubida	0.01	-0.03	0.05	0.02	-0.10	-0.15	-0.41	-0.33	-0.29	-0.11	0.14	0.86	1.06	0.36	0.09	0.11	0.16	0.01	-0.10	-0.10	-0.10	-0.08	-0.09

Autocorrelations of triatomine capture time series between *Triatoma gerstaeckeri* and each of the other most frequently collected triatomine species (*Triatoma sanguisuga*, *Triatoma indictiva*, *Triatoma lecticularia*, and *Triatoma rubida*) between –11 and 11 weeks. The analysis was restricted to the adult triatomines captured alive by citizen scientists. There were no *T. rubida* collected in 2013, only one *T. rubida* collected in 2014, only four *T. rubida* collected in 2015, and only six *T. indictiva* collected in 2016, which were insufficient for running the analysis. Color highlighting indicates cells with higher percentages.
